# Meetings of Societies

**Published:** 1927

**Authors:** 


### Meetings of Societies.

### Bristol Medico-Chirurgical Society.

*December 8th,* 1926.

Dr. A. L. Flemming, President, in the Chair.

Mr. D. A. Alexander read a paper entitled "**A Medical Year**" containing an interesting, but necessarily very condensed account of the many types of disease seen by a busy practitioner during that time and the speculations to which some of the cases gave rise.

Dr. J. V. Blachford read a paper on "**the Recommendations of the Royal Commission on Lunacy and Mental Disorder,**" which is published on p. 15.

In the discussion which followed, the President said that the slightness of the proposed changes was good evidence of the value of the present regulations. It had occurred to him to remark that many magistrates seemed to mistake their function, and to attempt to adjudicate on the medical evidence in the certificate, instead of seeing that legal procedure had been properly carried out.

Dr. E. Barton White said that he felt that, since it was inevitable that the law must step in where it was necessary to deprive a patient of his liberty, the provisional treatment order was a farce. The procedure proposed was exactly the same as that in force, except that the name "certification" was avoided. The stigma of the proposed method would be no less than that of the present, and the alteration would tend to crystallise the idea that certification implied disgrace, an idea that alienists had for years been labouring to dispel. As regards the voluntary patient, he felt that there was real risk of abuse under the scheme suggested, and that this risk would have to be met. The cost to institutions and to the public funds would certainly be increased by the new measures, for nearly every case would require to be certified for provisional treatment, many of these subsequently needing a second permanent certificate. The effect of the double procedure would be bad for many of the patients. The only valuable part of the report lay in the recommendation to increase the protection of those who had to deal with the insane.

Dr. J. R. P. Phillips agreed that the commission had failed to produce any useful recommendations. Extension of the voluntary system to the State-aided class was valuable, but the decision should lie with the family doctor and the medical superintendent concerned; without this the risk of abuse was too great. The proposed provisional order was exactly the same as a certificate. The report pandered to the old idea that sane persons are detained in asylums.

Dr. J. A. Nixon confirmed the view of the President that some magistrates had mistaken the function assigned to them. He quoted a conversation with the late Mr. F. A. Inderwick, K.C. who had assisted in drafting the Act of 1890. Mr. Inderwick had said that it was not intended that magistrates should attempt to decide the fact of insanity but should satisfy themselves that the certificates were in legal order and particularly that the certificates referred to the person about to be detained. "Many magistrates," Mr. Inderwick added, "arrogated to themselves the task of trying to decide whether the person was insane or not, and this the drafters of the Act had never supposed they would be foolish enough to attempt.

Dr. Blachford in the course of his reply to questions said that courses of instruction in clinics were of little value; the study and treatment of disorder of mind demanded the whole attention of a lifetime.

---

### Bristol and Bath Surgical Club.

The Autumn Meeting was held at Bath on December 4th, 1926, and was well attended. The programme was arranged by the President, Mr. Mumford, and opened with a clinical meeting at the Royal United Hospital. Two especially interesting specimens were a tumour of the femur, over which opinion was divided, and a retrograde intussusception of efferent jejunum into a gastro-enterostomy. In the tour of the wards we saw a patient apparently cured of epithelioma of the tonsil after diathermy. Mr. Mumford showed a beautiful example of plastic surgery by the use of a pedicle graft from the abdomen to the fore-arm. The treatment of various fractures provoked useful discussion. The Club later repaired to the Children's Hospital, where Miss M. Morris gave an instructive demonstration of methods of treatment of postural and other deformities. Although working with patients new to her, and severely deformed, she seemed to grasp their interest and from the outset to evoke a response.

